# Stop exsanguination by inflation: management of aorta-esophageal fistula bleeding

**DOI:** 10.1093/jscr/rjae120

**Published:** 2024-03-08

**Authors:** Kristina M Pagano, Alexander A Fokin, Michael Parra, Ivan Puente

**Affiliations:** Department of Surgery, Herbert Wertheim College of Medicine, Florida International University, 11200 SW 8th St AHC2, Miami, FL 33199, United States; Department of Trauma and Acute Care Surgery, Delray Medical Center, 5352 Linton Blvd, Delray Beach, FL 33484, United States; Department of Trauma and Acute Care Surgery, Broward County Health Care System, 1800 NW 49th Street, STE. 110, Fort Lauderdale, FL 33309, United States; Department of Trauma and Acute Care Surgery, Broward County Health Care System, 1800 NW 49th Street, STE. 110, Fort Lauderdale, FL 33309, United States

**Keywords:** aortoesophageal fistula, hematemesis, massive gastroesophageal bleeding, Sengstaken–Blakemore tube, REBOA, thoracic endovascular aortic repair

## Abstract

Aortoesophageal fistula is rare and typically presents itself to the emergency department as Chiari’s Triad of mid-thoracic pain, sentinel arterial hemorrhage, and exsanguination after a symptom-free interval. However, fatal bleeding may be the first and last presentation of an aortoesophageal fistula. When a patient experiences massive hematemesis without witnesses, EMS may assume that bleed is of a traumatic mechanism. We present a case of a 59-year-old male with no previous medical history who was transported to a trauma center unconscious and with massive bleeding of unknown origin. Computed tomography revealed a thoracic aortic aneurysm and an aortoesophageal fistula. Bleeding was not controlled and the patient expired. Trauma bay personnel should follow an algorithm which includes a prompt tamponade of the bleed using a Sengstaken–Blakemore tube or esophageal balloon paralleled by massive transfusion and obtaining an early computed tomography scan to manage patients with massive gastroesophageal bleeding until appropriate surgical interventions can be initiated.

## Introduction

An aortoesophageal fistula (AEF) is an abnormal connection between the aorta and the esophagus usually secondary to a thoracic aortic aneurysm (TAA) or foreign body ingestion. The presentation typically aligns with Chiari’s triad of mid-thoracic pain, sentinel arterial hemorrhage, and exsanguination after a symptom-free interval. Primary AEFs are very rare, with a reported incidence of 0.02% and 0.07% in autopsy studies [1]. Approximately 95% of patients with TAAs are asymptomatic before an acute event occurs [2]. Secondary AEFs occur at a relative incidence of 0.4%–2.4% [3] and are usually because of postoperative stenting complications. Primary and secondary AEFs are on the rise with the increase in aortic aneurysm diagnoses and stenting in exponentially increasing aging population, with almost 10% of Americans having some degree of aortic enlargement [4]. Despite the sequence of Chiari’s triad, fatal bleeding may be the first and last clinical presentation of the AEF. AEF with hematemesis is nearly always fatal but with appropriate treatment survival rates can be between 18% and 93% [5]. Therefore, prompt recognition and intervention is required. Computed tomography (CT) scan is the common modality for diagnosing an AEF. Esophagogastroduodenoscopy generally plays a role in the management of acute gastroesophageal bleeding, however, it can be dangerous as it may dislodge the thrombus and precipitate hemorrhage [6].

We present the case of a 59-year-old male who was delivered to a Level 1 trauma center for suspected traumatic bleeding but was ultimately diagnosed with a massive upper gastrointestinal bleed because of an AEF.

## Case report

**Figure 1:**
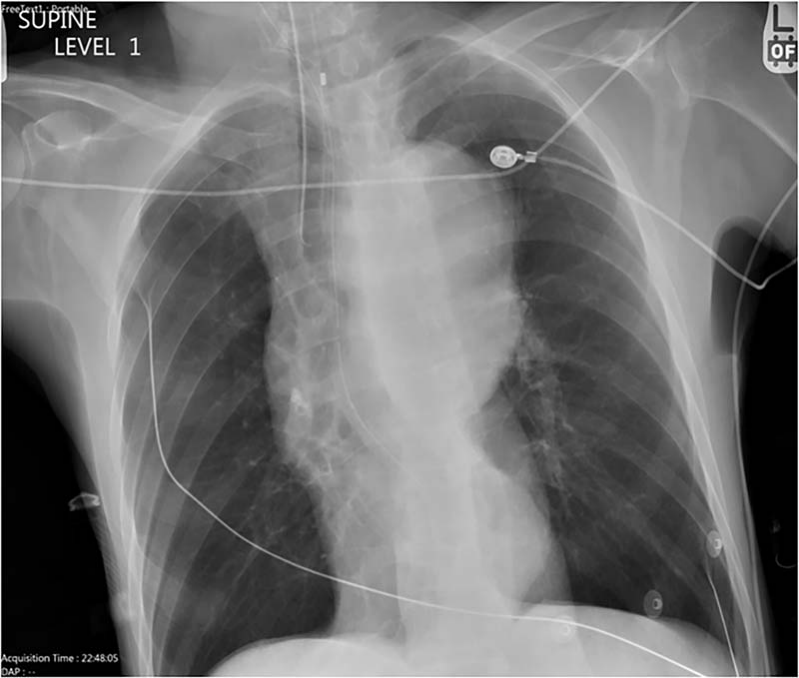
Chest X-ray with patient supine showing an impressive dilated thoracic aorta consistent with a large thoracic aneurysm.

**Figure 2:**
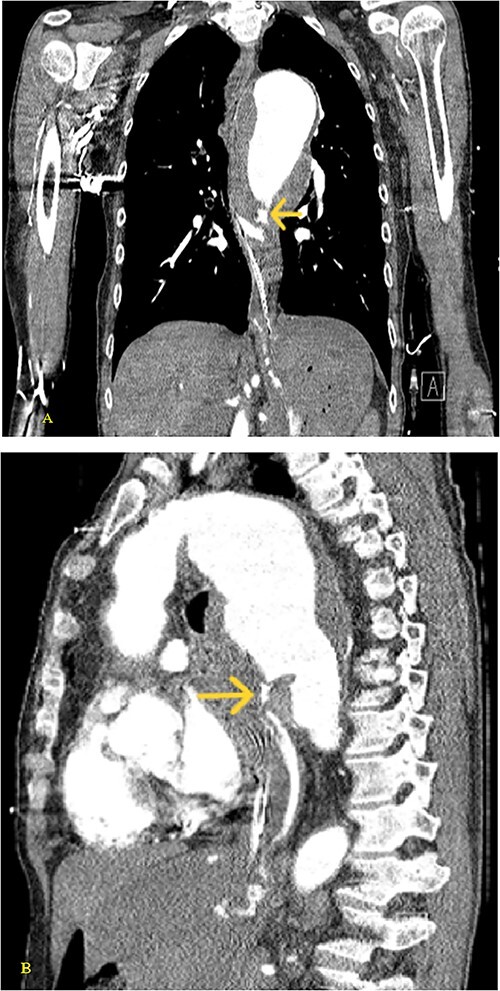
(A) Coronal and (B) sagittal views of the arterial phase CT chest with IV contrast performed during the initial trauma evaluation show aneurysmal dilation of the thoracic aorta with contrast extravasation into the esophagus and stomach.

**Figure 3:**
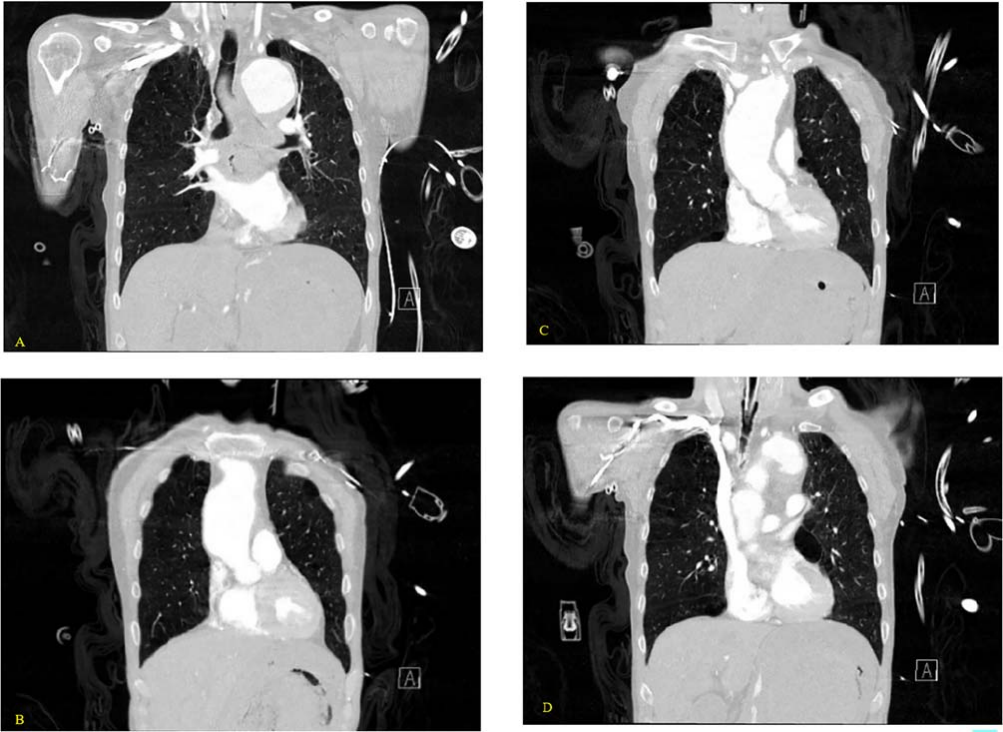
(A–D) Coronal CT chest with contrast reveal the descending aortic aneurysm of at least 6.5-cm diameter. Deep ulceration in the descending aorta at approximately the level of the pulmonary artery bifurcation. The ulceration has eroded into the esophagus. Extravasation of contrast is observed from the ulceration into the esophagus.

**Figure 4:**
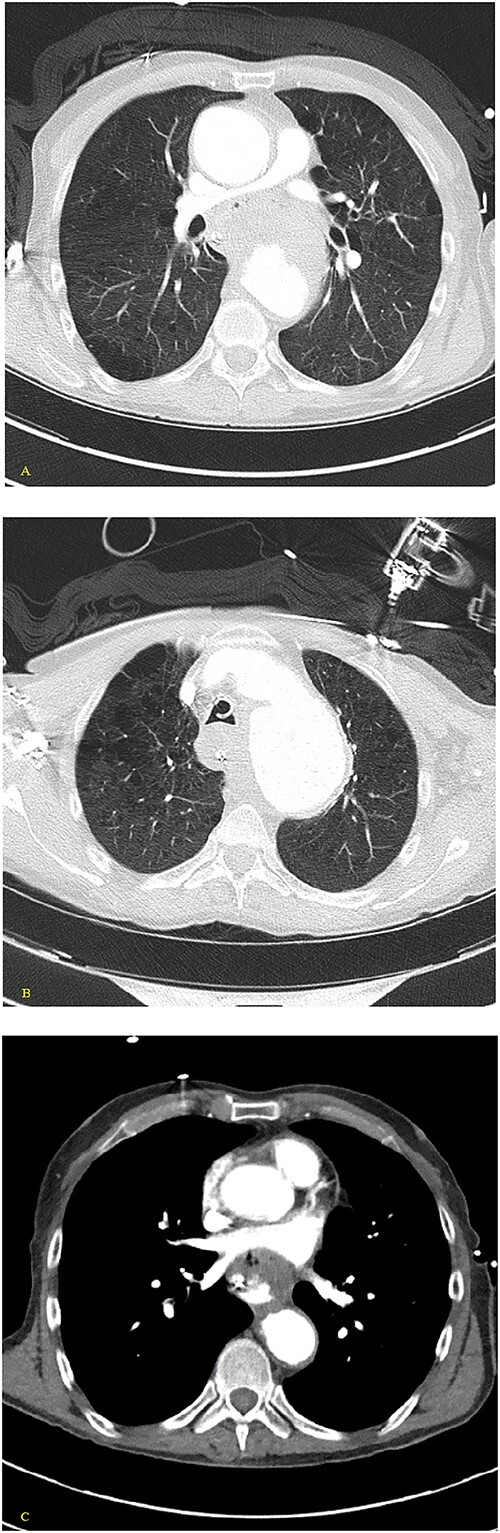
(A, B) Axial nonenhanced CT chest with contrast and (C) axial enhanced CT chest with contrast reveal an extensive clot around the aorta and around the esophagus with some mass effect on the pulmonary arteries and the LA. Clot fills the aorta.

A 59-year-old male with no known previous medical history presented to a Level 1 trauma center after being discovered by EMS in a large volume of red blood. The event was unwitnessed by bystanders, so it was assumed that the mechanism was a traumatic fall with a resulting head bleed. During transportation patient lost pulses but returned to spontaneous circulation after cardiopulmonary resuscitation. The patient’s initial vital signs in the trauma bay were a blood pressure of 129/40 mmHg, a heart rate of 101 beats/min, and a Glasgow Coma Score of 3. The head was bandaged by EMS with red blood sticking to the patient’s hair, ears, and face. Upon unwrapping of the patient’s bandaged head, there was no source of bleeding nor was any trauma identified to the body. The patient was intubated and an orogastric tube was inserted with immediate drainage of 2L of bright red blood. The initial differential diagnosis was trauma in origin, including a basilar skull fracture with ingestion of blood or blunt abdominal trauma. Sonography identified no signs of internal peritoneal bleeding. Chest X-ray revealed large dilation of the thoracic aorta (Fig. 1). CT of the chest with IV contrast showed aneurysmal dilation of the ascending and descending thoracic aorta with max diameter of 65 mm, AEF at the level of pulmonary artery bifurcation and contrast extravasation into the esophagus and stomach (Figs 2–4). Octreotide was given and a massive transfusion protocol (MTP) was initiated with transfusion including 2 units of whole blood and 15 units of RBCs. Cardiothoracic surgery and interventional radiology were called to evaluate the patient; however, massive blood loss ultimately led to a myocardial infarction and the patient expired 75 min after admission*.*

## Discussion

Massive hematemesis can be the first and last presentation of an AEF, whereas previous medical history can be unknown to the healthcare provider because of the patient being unconscious. EMS may assume a traumatic event seeing a massive pool of blood and can further obscure the diagnosis by bandaging the body parts and presenting the story that implies a traumatic mechanism for bleeding. With increasing rates of aortic aneurysms within our aging population, AEFs may preset to trauma personnel.

In all successful cases where massive upper gastroesophageal bleeding was stopped, an esophageal balloon [6, 7] or a Sengstaken–Blakemore tube (SBT) [8–10] was utilized, and this allowed for the temporary stabilization and had bought time for exact CT diagnosis and surgical intervention.

First, trauma bay personnel must be able to suspect the diagnosis of an AEF. Second, they must act fast with either SBT or esophageal balloon inflation to tamponade the bleed paralleled by MTP and prompt CT. These tools must be included in the trauma crash cart and personnel must be trained in their placement. Guidelines should be implemented at trauma centers to allow for this algorithmic approach for a suspected AEF and tamponade procedure. Upper gastrointestinal bleeding from other etiologies such as Budd–Chiari syndrome [11] and esophageal varices [12] continue to increase in its incidence, so the same algorithm can be applied.

Other candidates for tamponade include the use of a resuscitative endovascular balloon occlusion of the aorta (REBOA) [13, 14]. The aorta Zone 1 is the most common location for aortic aneurysms and subsequent AEFs. Using REBOA in this location for a prolonged period is associated with severe complications [13]. The ideal position for the balloon is at the fistula level, which can be placed using the esophageal balloon, but with REBOA, it must be inflated above the upper tip of the aneurysmatic sac. The compromised morphology of the aortic wall associated with aneurysms is still of concern and REBOA placement may potentially lead to a dissection. Also, REBOA is not realistic at the aortic arch. On the contrary, the SBT or esophageal balloon is very fast and easy to use. Their use in tandem with MTP to stabilize the patient will allow time for a vascular-trained surgeon or interventional radiology to initiate thoracic endovascular aortic repair (TEVAR) or open surgery for the best chances of survival [15].

## Conclusion

The need for an algorithmic approach to prompt stabilization and diagnoses is vital for the prevention of fatality in cases of AEF bleed. Application of either a SBT or esophageal balloon while providing MTP and CT can be the means to increase survival of patients who present to the trauma bay with suspected AEF and massive upper gastroesophageal bleeding and can allow for further intervention to be pursued such TEVAR or open surgery.
